# 

**Published:** 1880-08

**Authors:** 


### HYMENEAL.

"Domestic happiness, thou only bliss
Of paradise that has survived the fall."

On June 30, at Woodside, in Loudoun county, Virginia, at the home of the bride, by the Rev. Arthur Johns, Dr. Francis L. Galt of Norfolk, to Lucy H. Randolph, daughter of the late James J. Randolph, Esq.

### NECROLOGICAL.

"Death is the crown of life:
Were death denied, poor man would live in vain."

Died on June 14th ultimo, at his residence, Pelham, Westchester county, N. Y., Dr. Richard Lewis Morris, in the 75th year of his age.

*Prospectus of the*

### Independent Practitioner:

### A MONTHLY JOURNAL.

*DEVOTED TO MEDICAL, SURGICAL, OBSTETRICAL, DENTAL AND HYGIENICAL SCIENCE.*

The fixed purpose of the projectors of this journal to adhere with unvarying fidelity to independence in all matters appertaining to its conduct and management, renders it unnecessary to issue a lengthy prospectus of its objects and aims. With a few brief statements, therefore, it will be launched forth upon the sea of journalism to take its chances for public favor among the many craft now sailing more or less propitiously thereon. While it respectfully invites subscriptions from every quarter, it naturally looks for primary assistance from old friends and acquaintances. It will know no sectionalism, and, therefore, confidently expects to merit the friendship and good will of those who *really* desire to sustain and keep from degredation to the level of the merest trade, the grandest and noblest calling known to humanity. From all such, subscriptions, contributions on subjects of interest to the profession, and advertisements are respectfully solicited. The experience of the senior editor, in the past, will be brought into activity in catering to the taste of its readers, and nothing will ever mar its pages not consistent with the standard that has been erected for its conduct and management. Being entirely unconnected with colleges and cliques, it will be bold, fearless and honest in speaking and upholding the right in *all* matters coming within its purview, and will scrupulously render even-handed justice to every one. All subjects of interest to Physicians, Obstetricians, Surgeons and Dentists will claim the attention of its editors and find space in its pages. To this end translations from the French and German periodicals will be introduced from time to time, and the English and American journals made tributary to its columns. All necessary arrangements have been made to *secure permanency* to the enterprise, and to keep it the independent echo and exponent of all that is new and good in the field of its labors. Exchanges of Medical, Dental and other scientific journals are respectfully requested from all parts of the world. Exchanges and books, except Dental, for notice or review, should be addressed to the senior editor, and Dental exchanges, subscriptions, advertisements, and *all* matters relating to the business management of the journal, should be sent invariably to the junior editor and proprietor.

HARVEY L. BYRD, A. M., M. D.,

*No. 147 Edmondson Avenue.*

BASIL M. WILKERSON, D. D. S., M. D.,

*No. 68 N. Charles Street.*

WILL *YOU* PLEASE SEND IN *YOUR* SUBSCRIPTION MONEY NOW?

Many persons who receive pamphlets, papers, etc., through the mails are unaware of the United States laws relating to them, and such will feel indebted to us for the information and guidance the enactments will afford which we copy below, viz:

1. A postmaster is required to give notice *by letter* (returning a paper does not answer the law) when a subscriber does not take his paper out of the office, and state the reason for its not being taken. Any neglect to do so makes the postmaster *responsible* to the publishers for payment.

2. Any person who takes a paper from the post-office, whether directed to his name or another, or whether he has subscribed or not, is responsible for the pay.

3. If a person orders his paper discontinued, he must pay all arrearges, or the publisher may continue to send it until payment is made, and collect the whole amount, *whether it be taken from the office or not.* There can be no legal discontinuance until the payment is made.

4. If the subscriber orders his paper to be stopped at a certain time, and the publisher continue to send, the subscriber is bound to pay for it *if he takes it out of the post-office.* The law proceeds upon the ground that a man must pay for what he uses.

5. The courts have decided that refusing to take a newspaper and periodicals from the post-office, or removing and leaving them uncalled for, is *prima facie* evidence of intentional fraud.

This column will be sold for advertising space six months during the year at *special rates.*

N. B.—Each month THE INDEPENDENT PRACTITIONER will be received by a large number of persons for the *first* time, all of whom we trust will be induced to become subscribers. They will please remit NOW the amount of subscription ($2.00) to insure the continuation of the journal. Money can be sent at our risk by *post-office money order*, registered letter or checks on banks in *N. Y. City or Baltimore, made payable to Dr. B. M. Wilkerson.* We hope that the few who have been receiving the journal and have not "paid up" will cheerfully do so *now.*

